# Supplementary material for: Effect of high-flow nasal therapy on patient-centred outcomes in patients at high risk of postoperative pulmonary complications after cardiac surgery: update to the statistical analysis plan for NOTACS, a multicentre adaptive randomised controlled trial
Source: Trials. 2024 Nov 6;25:741. doi: 10.1186/s13063-024-08538-3 (PMC11539829; doi:10.1186/s13063-024-08538-3)
Supplement: Supplementary file 1 — Supplementary Material 1. [file 13063_2024_8538_MOESM1_ESM.docx]

**Appendix 1**

The NOTACS study team members:

Table 1. List of NOTACS investigators

| **Name** | **Role** | **Affiliation** |
| --- | --- | --- |
| **UK** | | |
| Andrew Klein | Chief Investigator; Consultant Anaesthetist | Department of Anaesthesia, Royal Papworth Hospital |
| Jo Steele | Senior Data Manager | Papworth Trials Unit Collaboration, Royal Papworth Hospital |
| Ayman Mohammed | Data Manager | Papworth Trials Unit Collaboration, Royal Papworth Hospital |
| Ellen Temple | Clinical Trial Manager | Papworth Trials Unit Collaboration, Royal Papworth Hospital |
| Melissa Duckworth | Clinical Project Manager | Papworth Trials Unit Collaboration, Royal Papworth Hospital |
| Fiona Bottrill | Study Monitor; QA Project Manager | Papworth Trials Unit Collaboration, Royal Papworth Hospital |
| Sofia Villar | Senior Statistician; MRC Investigator - Programme Leader | MRC Biostatistics Unit, University of Cambridge |
| Sarah Dawson | Study Statistician | MRC Biostatistics Unit, University of Cambridge |
| Julia Fox-Rushby | Senior Health Economist | King’s College London, London, UK |
| Siddesh Shetty | Study Health Economist | King’s College London, London, UK |
| Gudrun Kunst | Consultant Anaesthetist & Professor of Cardiovascular Anaesthesia | Department of Anaesthetics and Pain Therapy, King’s College Hospital NHS FT, London; School of Cardiovascular and Metabolic Medicine & Sciences, King’s College London |
| Gavin J Murphy | Academic Cardiac Surgeon; Director of Leicester Clinical Trials Unit | Leicester NIHR Biomedical Research Centre |
| Guillermo Martinez | Consultant Anaesthetist | Department of Anaesthesiology, Royal Papworth Hospital NHS Foundation Trust |
| Carol Freeman | Clinical Project Manager (former) | Papworth Trials Unit Collaboration, Royal Papworth Hospital |
| Melissa Earwaker | Trial Manager (former) | Papworth Trials Unit Collaboration, Royal Papworth Hospital |
| Yi-Da Chiu | Study Statistician (former) | Papworth Trials Unit Collaboration, Royal Papworth Hospital |
| Vasileios Zochios | Consultant in Adult ECMO and Intensive Care Medicine | Cardiothoracic Critical Care, Glenfield Hospital, University Hospitals Leicester |
| Val Bussey | PPI Representative (former, withdrew) | NA |
| Robin McClean | PPI Representative (former, withdrew) | NA |
| Geoff Brown | PPI Representative (former, deceased) | NA |
| **Australia** | | |
| A/Prof. Ed Litton | Chief Investigator Australia; Head of ICU Research | Curtin School of Population Health, Curtin University, Fiona Stanley Hospital, Perth Western Australia |
| Dr. Julieann Coombes | Australian Management Co-committee Chair; Program Lead, Indigenous Methodologies and Systems Change, Guunu-maana (Heal) Aboriginal & Torres Strait Islander Health | The George Institute for Global Health, Australia |
| Camila Kairuz Santos | Australian sub-study support | The George Institute for Global Health, Australia |
| Prof David Pilcher | Principal Investigator; Senior Intensive Care Specialist; Adjunct Clinical Professor | Department of Intensive Care, The Alfred Hospital, Melbourne; The Australian and New Zealand Intensive Care – Research Centre, Monash University, Melbourne, Victoria |
| Prof Andrew Maiorana | Professor of Clinical Exercise Physiology; Head of the Department of Exercise Physiology | Curtin School of Allied Health, Curtin University; Fiona Stanley Hospital, Perth, Western Australia |
| Prof Christopher Reid | John Curtin Distinguished Professor; Cardiovascular Epidemiologist | School of Public Health and Preventive Medicine, Monash University; Curtin School of Population Health, Curtin University, Perth, Western Australia |
| Dr Sumit Yadav | Specialist Cardiothoracic Surgeon; Senior Lecturer | Mater Private Hospital Townsville; James Cook University, Townsville, Queensland |
| Dr Mahesh Ramanan | Principal Investigator; Staff Specialist in Intensive Care Medicine | The Prince Charles Hospital, Brisbane, Australia, Brisbane, Queensland |
| Dr Siva Senthuran | Principal Investigator; Senior Specialist in Intensive Care Medicine | Townsville University Hospital, Australia, Townsville, Queensland |
| Keziah Bennett-Brook | Program Head of the Guunu-maana (Heal) Aboriginal and Torres Strait Islander Health Program | The George Institute for Global Health, Australia, NSW |
| Prof Anthony Delaney | Principal Investigator; Senior Staff Specialist; Associate Professor | Malcolm Fisher Department of Intensive Care Medicine, Royal North Shore Hospital; Faculty of Medicine and Health, The University of Sydney, NSW |
| A/Prof Tamara Mackean | Associate Professor; Senior Research Fellow | College of Medicine and Public Health, Flinders University, Adelaide, South Australia; The George Institute for Global Health, NSW |
| Prof Neil Orford | Principal Investigator; Senior Staff Specialist in Intensive Care; Associate Professor of Intensive Care Medicine | University Hospital Geelong; Deakin University School of Medicine; Geelong, Victoria |
| Dr Lavinia Tran | Research Fellow | Centre of Cardiovascular Research and Education in Therapeutics (CCRET), Monash University, Melbourne, Victoria |
| Dr Sananta Dash | Principal Investigator Staff Specialist of Intensive Care | Townsville University Hospital, Townsville, Queensland |
| Dr Matthew Maiden | Senior Specialist – Intensive Care Medicine; Associate Professor | The Royal Melbourne Hospital, Victoria, Australia; Department of Critical Care, University of Melbourne, Melbourne, Victoria |
| Jacquita Affandi | NOTACS Project Manager Australia | Curtin University and Fiona Stanley Hospital, Australia |
| **New Zealand** | | |
| A/Prof Rachael Parke | Principal Investigator, Associate Professor; Nurse Senior Research Fellow | School of Nursing, University of Auckland; Cardiothoracic and Vascular ICU at Auckland City Hospital, Auckland, New Zealand |
| Dr Shay McGuinness | Principal Investigator; Intensive Care Specialist | Cardiothoracic and Vascular ICU at Auckland City Hospital, Auckland, New Zealand |

Table 2. List of NOTACS Trial Steering Committee members

| **Name** | **Role** | **Affiliation** |
| --- | --- | --- |
| Prof Susan Griffin | Independent Health Economist; Professor of Health Economics | Centre for Health Economics, University of York |
| Prof Stephen Brett | Independent Chair; Professor of Critical Care; Consultant in Intensive Care Medicine | Dept of Surgery and Cancer, Imperial College London; Imperial College Healthcare NHS Trust |
| Dr Philip Pallmann | Independent Statistician; Senior Research Fellow (Statistics) | Centre for Trials Research,  College of Biomedical and Life Sciences,  Cardiff University |
| Dr Peter Shirley | Independent Clinician; Consultant in Intensive Care Medicine and Anaesthesia | Royal London Hospital, Barts Health NHS Trust |
| Andrew Hoppington | PPI Representative | N/A |
| Marijcke Veltman | PPI Representative | N/A |

Table 3. List of NOTACS Data Monitoring and Ethics Committee (DMEC) members

| **Name** | **Role** | **Affiliation** |
| --- | --- | --- |
| Prof Mahmoud Loubani | Independent Chair | Hull University Teaching Hospitals NHS Trust |
| Prof Graeme MacLennan | Independent Statistician | University of Aberdeen |
| Dr Peter Alston | Independent Cardiac Anaesthetist | Royal Infirmary of Edinburgh |
| Prof Thomas Jaki | Independent Statistician  (former) | Lancaster University |

Table 4. List of participating sites in the NOTACS trial

| **Country** | **Study Site** |
| --- | --- |
| United Kingdom | - Royal Papworth Hospital, Royal Papworth Hospital NHSFoundation Trust - Royal Brompton Hospital, Guy's and St Thomas' NHS Foundation Trust - King’s College Hospital, King's College Hospital NHS Foundation Trust - University Hospital of Wales, Cardiff and Vale University Local Health Board - Golden Jubilee University National Hospital, NHS Golden Jubilee - St Thomas’ Hospital, Guy's and St Thomas' NHS Foundation Trust - Glenfield Hospital, University Hospitals of Leicester NHS Trust - James Cook Hospital, South Tees Hospitals NHS Foundation Trust - Queen Elizabeth Hospital, University Hospitals Birmingham |
| **Australia** | - St John of God Geelong Hospital, St John of God Healthcare - The Prince Charles Hospital, Metro North Health - Fiona Stanley Hospital, South Metropolitan Health Service - The Townsville Hospital, Townsville Hospital and Health Service - Royal North Shore Hospital, Northern Sydney Local Health District - The Alfred Hospital, Alfred Health - University Hospital Geelong, Barwon Health |
| **New Zealand** | - Auckland City Hospital, Te Whatu Ora – Health New Zealand |
